# Strengthening of Surveillance during Monkeypox Outbreak, Republic of the Congo, 2017

**DOI:** 10.3201/eid2406.180248

**Published:** 2018-06

**Authors:** Reena H. Doshi, Sarah Anne J. Guagliardo, Angelie Dzabatou-Babeaux, Camille Likouayoulou, Nestor Ndakala, Cynthia Moses, Victoria Olson, Andrea M. McCollum, Brett W. Petersen

**Affiliations:** Centers for Disease Control and Prevention, Atlanta, Georgia, USA (R.H. Doshi, S.A.J. Guagliardo, V. Olson, A.M. McCollum, B.W. Petersen);; Ministry of Health, Brazzaville, Republic of the Congo (A. Dzabatou-Babeaux, C. Likouayoulou);; Kinshasa School of Public Health, Kinshasa, Democratic Republic of the Congo (N. Ndakala);; International Communication and Education Foundation, Kinshasa (C. Moses)

**Keywords:** monkeypox, monkeypox virus, viruses, outbreak, surveillance, epidemiology, healthcare workers, community members, zoonoses, Likouala Department, Republic of the Congo

## Abstract

Reports of 10 suspected cases of monkeypox in Likouala Department, Republic of the Congo, triggered an investigation and response in March 2017 that included community education and surveillance strengthening. Increasing numbers of outbreaks suggest that monkeypox virus is becoming a more prevalent human pathogen. Diverse approaches are necessary for disease control and prevention.

On January 27, 2017, the Republic of the Congo Division of Disease Control was notified of 2 suspected human cases of monkeypox (MPX) in Likouala Department, in the northern part of the country, which prompted a local investigation. In March 2017, after 8 additional suspected cases were reported, the Republic of the Congo Division of Disease Control joined with external partners (World Health Organization, United Nations High Commissioner for Refugees, US Centers for Disease Control and Prevention) and 2 Field Epidemiology and Laboratory Training Program trainees from the neighboring Democratic Republic of the Congo (DRC) to investigate suspected cases and strengthen epidemiologic surveillance in the region.

Although human cases of MPX are routinely reported in the DRC (*1*,*2*), cases are only sporadically reported in the Republic of the Congo; large outbreaks previously occurred in 2003 and 2011 in Likouala Department (*3*,*4*). Poor transportation and communication infrastructure in the region, in addition to competing public health priorities, have contributed to a paucity of knowledge among healthcare workers (HCWs) about MPX case recognition, notification, and reporting. Local HCWs unofficially report MPX, but inconsistent and incomplete case notifications continue to be a challenge.

MPX, a zoonotic orthopoxvirus, is a public health priority in regions of endemicity in West and Central Africa because of its clinical severity and potential for epidemic spread (*1*). The virus is a member of the same genus as variola virus, and the clinical presentation of MPX resembles that of smallpox, with the addition of lymphadenopathy (*5*). Symptoms include an initial febrile prodrome (1–4 days), followed by a disseminated vesiculopustular rash, which includes the palms of the hands and soles of the feet (*6*). Transmission occurs through contact with infectious lesions, contaminated fomites, or respiratory droplets (believed to be most common for human-to-human transmission) (*6*). When human-to-human transmission occurs, identification of persons who have had extensive contact with a MPX patient is critical to limit the spread of disease and prevent outbreaks.

During March 15–22, 2017, a total of 139 HCWs were trained in 7 towns throughout the study region. HCWs received training in MPX clinical characteristics and case recognition; case management; surveillance; and infection prevention and control, including donning and removal of personal protective equipment. Content for the training materials was derived from a 2000 World Health Organization MPX manual with contributions from subject matter experts, further revised after a similar training was conducted in DRC in 2010 (*7*). In addition, HCWs were provided with MPX investigation kits that included surveillance manuals, MPX-specific case investigation forms (which collect demographic, clinical, and exposure information), personal protective equipment, and sample collection supplies to enhance laboratory-based surveillance.

A nongovernmental organization (International Communication and Education Foundation, Homestead, FL, USA) provided community outreach and education. Educators from this organization held screenings of short films in Lingala (the local language) featuring families who had experienced MPX and local public health officials. The educational films were designed to be interactive in nature; community members were encouraged to discuss, debate, and ultimately develop prevention mechanisms/lifestyle changes that will result in zoonotic disease prevention. Educators held screenings in 14 villages in Likouala Department and educated >1,160 community members.

During January–December 1, 2017, a total of 81 suspected MPX cases, 7 laboratory-confirmed cases, and 6 deaths from this disease were reported in Likouala Department. Outbreaks of measles and infection with varicella zoster virus, which are often confused with MPX virus infections, were reported in the region before and during the investigation period. Thus, it was difficult to determine if this is a true increase, an artifact of strengthened surveillance in March, or merely the endemic rate of MPX in the region.

Although enhancing disease surveillance was a priority during the outbreak, there remain numerous challenges to consistent MPX reporting. The Republic of the Congo lacks specific programs to adequately train and support HCWs, and capacity is hindered by the need to cover vast, inaccessible areas that have underdeveloped infrastructure and limited resources. Leveraging resources and reinforcing HCW capacity through ongoing training at the local level will be vital for improving surveillance and effectively responding to outbreaks in the area. Implementation of a surveillance program modeled in a manner similar to other MPX-endemic countries (such as the DRC) could be useful (*8*). In the absence of consistent laboratory diagnostics, detection of endemic MPX cases will require a more specific surveillance case definition (*9*). In addition, investing in training programs, such as the Field Epidemiology and Laboratory Training Program, could provide increased support. Finally, standardizing a multifaceted response that includes community education, for other countries where MPX outbreaks are most likely to occur, such as the DRC, Cameroon, and most recently, Nigeria, could be extremely useful.
